# Cu(II) and Ni(II) Interactions with the Terminally Blocked Hexapeptide Ac-Leu-Ala-His-Tyr-Asn-Lys-amide Model of Histone H2B (80–85)

**DOI:** 10.1155/2008/257038

**Published:** 2008-04-13

**Authors:** Katerina Panagiotou, Maria Panagopoulou, Tilemachos Karavelas, Vassiliki Dokorou, Andrew Hagarman, Jonathan Soffer, Reinhard Schweitzer-Stenner, Gerasimos Malandrinos, Nick Hadjiliadis

**Affiliations:** ^1^Department of Chemistry, University of Ioannina, 45110 Ioannina, Greece; ^2^Department of Chemistry, Drexel University, 3141 Chestnut Street, Philadelphia, PA 19104, USA

## Abstract

The N- and C-terminal blocked hexapeptide Ac-Leu-Ala-His-Tyr-Asn-Lys-amide (LAHYNK) representing the 80–85 fragment of histone H2B was synthesized and its interactions with Cu(II) and Ni(II) ions were studied by potentiometric, UV-Vis, CD, EPR, and NMR spectroscopic techniques in solution. Our data reveal that the imidazole N(3) nitrogen atom is the primary ligating group for both metal ions. Sequential amide groups deprotonation and subsequent coordination to metal ions indicated an {N_imidazole_, 3N_amide_} coordination mode above pH∼9, in all cases. In the case of Cu(II)-peptide system, the almost exclusive formation of the predominant species CuL in neutral media accounting for almost 98% of the total metal ion concentration at pH 7.3 strongly indicates that at physiological pH values the sequence -LAHYNK- of histone H2B provides very efficient binding sites for metal ions. The imidazole pyrrole N(1) ionization (but not coordination) was also detected in species CuH_−4_L present in solution above pH ∼ 11.

## 1. INTRODUCTION

In eukaryotes, the genome is organized as a highly complex and condensed structure called chromatin, the unit of which is the nucleosome. It is well known that about 146-147 DNA base pairs are bound around a histone octamer which contains two copies of each of histones H2A, H2B, H3, and H4, forming the nucleosomes [1]. Chemical and structural description of the possible metal binding sites inside the cell nuclei could provide the molecular basis for a better understanding of the interactions responsible for cancer or similar genetic disease developments. It is believed that the binding abilities of the metal ions inside the cell nuclei may have repercussions in the genetic code. Having in mind the efficiency of metal ion coordination to proteins and DNA and the plenitude of histones into the nucleosome core, it is assumed that histones can be the fundamental binding sites for metal ions.

In this respect, previous studies have shown that certain histone peptide model, fragments of H2A, H2B, H3, and H4 may serve as efficient binding sites for metal ions such as Cu(II) and Ni(II) [2–14]. Moreover, interactions of metal ions with small peptides have revealed that His residues are the major coordination sites of several transition metal ions, including Ni(II) and Cu(II), in proteins [15–18].

Our research group initial studies in this interesting area, included the coordination abilities of Ni(II) and Cu(II) ions towards a series of terminally blocked hexapeptide models of the C-terminal “tail” -ESHH- of histone H2A. It was found that Ni(II) ions promoted hydrolysis of the peptides -TASHHK- and -TESAHK- at the -X-Ser- sites and this was assigned to the presence of the Ser residue [6–8, 10, 13] with an –OH group near the coordination site.

The driving force of the hydrolysis reaction of H2A histone blocked hexapeptide models was the high thermodynamic stability of Ni(II) or Cu(II) complexes with the hydrolysis products -SHHK- and -SAHK-, respectively [10]. Continuing our work, we have recently reported the efficient binding of the blocked hexapeptide -ELAKHA- model of the C-terminal of histone H2B to Cu(II) and Ni(II) ions [11], and in a more comprehensive study [14] the binding abilities of the C-terminal 71–76 fragment of histone H4 Ac-ThrTyrThrGluHisAla-amide in which Ni(II) mediated hydrolysis of the peptide bond Tyr-Thr was evident [14], in agreement with previous findings concerning the hydrolytic cleavage of the peptide bond Glu-Thr of the peptide -TETHHK- [9].

**Scheme 1:**
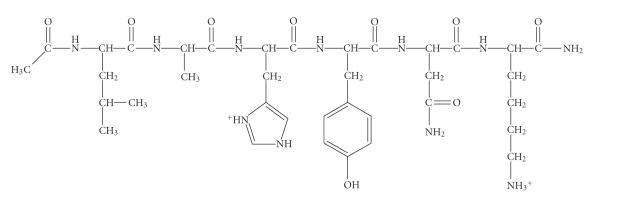
The hexapeptide Ac-Leu-Ala-His-Tyr-Asn-Lys-amide (-LAHYNK-).

Taking into account, the variety of possible coordination sites of histones plus the great anchoring capability of the histidyl residues towards Cu(II) and Ni(II) ions as stated above, we decided to continue our investigation with the new hexapeptide model -LAHYNK-, the 80–85 fragment of histone H2B (Scheme 1) which, according to the X-ray crystal structure of the nucleosome core [1], may be accessible for metal ions binding.

To make the peptide a more reliable model of the H2B 80–85 protein sequence, N- and C-termini were blocked by acetylation and amidation, respectively. A combination of potentiometric and spectroscopic (UV-Vis, CD, EPR, NMR) studies were applied in order to evaluate the formation models, species stoichiometry and possible coordination modes reported in this work.

## 2. EXPERIMENTAL

### 2.1. Materials

Trifluoroethanol (TFE), 1-hydroxybenzotriazole (1-HOBt), anisole, trifluoroacetic acid (TFA), 3-(trimethylsilyl)propionic acid sodium salt (TSP), D_2_O, and DCl were purchased from Sigma-Aldrich Chemical Co. (Milwaukee, Wis, USA). Cu(NO_3_)_2_ ⋅ 6H_2_O, Ni(NO_3_)_2_ ⋅ 6H_2_O, HNO_3_, KNO_3_, acetonitrile (HPLC grade), dicyclohexylcarbodiimide (DCC), ethanediol, HCl, and KOH were obtained from E. Merck (Darmstadt, Germany). Diethyl ether, isopropanol, dimethylformamide, and dichloromethane were purchased (analytical grade) from Labscan Chemical Co. (Dublin, Ireland). The resin H-Linker-2-chlorotrityl and the protected amino acids, Fmoc-His(*Mtt*)-OH, Fmoc-Lys(*Boc*)-OH, Fmoc-Leu-OH, Fmoc-Asn-OH, Fmoc-Tyr(OBu^*t*^), and Fmoc-Ala-OH, were purchased from CBL Chemicals Ltd. (Patras, Greece).

### 2.2. Methods

#### 2.2.1. Peptide synthesis

The blocked hexapeptide Ac-Leu-Ala-His-Tyr-Asn-Lys-amide (-LAHYNK-) (Scheme 1) was synthesized in the solid phase using the Fmoc chemistry [19, 20]. 1-hydroxybenzotriazole (1-HOBt) and dicyclohexylcarbodiimide (DCC) were used as coupling reagents, where Fmoc protection groups were removed before each coupling step by a 25% (v/v) piperidine solution in DMF. The removal of the protecting groups *Mtt-*, *Boc-*, and *Bu^t^-* was accomplished by a 65% TFA in CH_2_Cl_2_:TFE (6:1 v/v) solution and 5% anisole, as described in the literature [19, 20]. The peptide cleavage from the resin was performed using a CH_2_Cl_2_:TFE:CH_3_COOH (7:2:1 v/v) solution. The crude peptide was purified by preparative HPLC (Dionex system equipped with a P 580 pump) on a Waters C_18_ column (300×9 mm, 15 *μ*m, 300 Å) and its purity was finally confirmed by means of one-/two-dimensional ^1^H-NMR techniques (Varian Unity 500 MHz).

#### 2.2.2. Potentiometry

Potentiometric titrations of free hexapeptide and its complexes with Cu(II) and Ni(II) ions were carried out at 25°C, using a total volume of 2.0 mL. The titrations were performed in the presence of 0.1 M KNO_3_ over the pH range 2.5–11 on a MOLSPIN pH-meter system (Molspin automatic titrator, Molspin Ltd., Newcastle-upon-Tyne, U.K.), using a 0.500 mL micrometer syringe and a combined glass-silver chloride electrode calibrated in hydrogen concentrations using HNO_3_ [21]. 0.1 M KOH was used as titrant. The concentrations of the hexapeptide and the metal ions were between 2 mM and 1 mM at metal:peptide molar ratio between 1 : 1 and 1 : 2. For the binary system -LAHYNK-/Ni(II), excess of the hexapeptide (1 : 2) had been used to achieve shorter equilibrium times, due to the slow kinetics of the formation of the square-planar nickel complexes at a ratio 1 : 1. The experimental data were analyzed using the HYPERQUAD program [22]. Standard deviations computed by HYPERQUAD refer to random errors.

#### 2.2.3. NMR spectroscopy

NMR experiments were performed on a Varian Unity spectrometer at 500 MHz. The one-dimensional ^1^H-NMR experiments were performed in 99.9% D_2_O solutions at a peptide concentration of 7 mM, (pH* 9.5 at 25°C) in the absence or presence of Ni(II) ions (metal:peptide molar ratio 1 : 2). It must be mentioned that the pH* reading of the electrode was not corrected for the isotope effect. Additionally, ^1^H-^1^H-TOCSY experiments were used to assign the one-dimensional spectra of both free- and Ni(II)-bound peptide.

#### 2.2.4. CD spectroscopy

CD spectra were recorded on a Jasco J-810 spectropolarimeter, in the spectral range of 190–800 nm and pH range 3–12, in 0.1 cm cuvettes, using 2 mM of Cu(II) and 2.5 mM of Ni(II) ions. The used Cu(II):peptide and Ni(II):peptide molar ratios were 1 : 1.1 and 1 : 2, respectively. The temperature was controlled by a Peltier heating/cooling system from 5° −90°C (278–363 K) (±0.1°C). Ten-twenty accumulations were averaged using a 5 nm band width, a 500 nm/min scanning speed, and a 0.1 or 0.5 nm data pitch. Additionally, a background subtraction was carried out for all the spectra using similar parameters.

#### 2.2.5. UV/Vis spectroscopy

The absorption spectra of the complexes were recorded on a Jenway 6400 spectrophotometer in the spectral range of 320–900 nm, using 1 cm cuvettes. The spectra were recorded over pH range 3.5–11 by adding small amounts of concentrated KOH solution manually. Changes of the pH were monitored by a combined glass-silver chloride electrode. The used Cu(II):peptide and Ni(II):peptide ratios were 1 : 1.1 and 1 : 2, respectively.

#### 2.2.6. EPR spectroscopy

The X-band EPR spectra were obtained at 120 K on a Varian E109 spectrometer, using 4 mM of hexapeptide at Cu(II):peptide molar ratio 1 : 1.1. Ethanediol aqueous solution (20% v/v) was used as a solvent to ensure homogeneity of the frozen samples.

## 3. RESULTS AND DISCUSSION

### 3.1. Peptide characterization and protonation constants

Our peptide -LAHYNK- (Scheme 1) was acetylated and amidated in the N- and C-terminal, respectively in order to simulate a more realistic model of the histone H2B 80–85 sequence and it was characterized by means of NMR spectroscopy. NMR chemical shift values are presented in Table 1.

Overall protonation and ionization constants (p*K*_*a*_) derived by the potentiometric measurements on the free ligand are collected in Table 2.

The peptide can be considered as a triprotic acid (H_3_L) due to the presence of three ionizable groups in the measurable pH range (2–12), which are, the imidazole N(3) atom of histidine, the phenolic hydroxyl group, and the *ε*-amino group of the Lys side chain. The first p*K*_*a*_ value 6.23 is assigned to imidazole N(3)-H ionization while the next two (9.52, 10.53) to Tyr-phenolate, and lys-*ε*-NH_2_ groups, respectively. The above data are in good agreement with literature values corresponding to the above ionization types [7, 23, 24].

### 3.2. Cu(II) complexes

**Figure 1:**
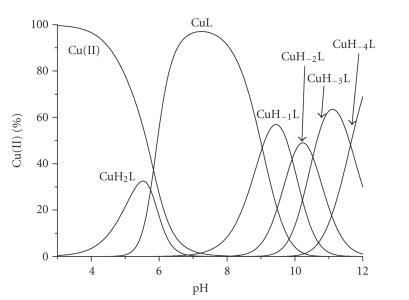
Species distribution diagram of Cu(II) complexes with -LAHYNK- (1 : 1), C_peptide_ = 2 mM.

The interaction of Cu(II) and the hexapeptide was studied in aqueous solutions in 1 : 1 and 1 : 2 molar ratios. The titration curves were best fitted assuming the formation of six species over the pH range 3–12, namely, CuH_2_L, CuL, CuH_−1_L, CuH_−2_L, CuH_−3_L, and CuH_−4_L. The stability constants of the above Cu(II) complexes with the hexapeptide -LAHYNK- are presented in Table 3 and the species distribution diagram of Cu(II) complexes is presented in Figure 1.

**Figure 2:**
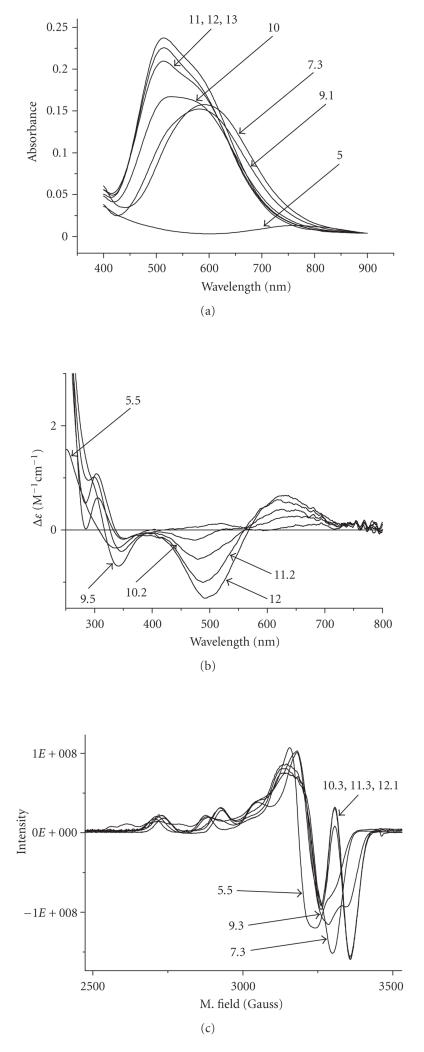
(a) UV/Vis, (b) CD and (c) EPR spectra of the system Cu(II)/-LAHYNK- (1 : 1) at various pH values.

For the assignment of the species UV/Vis, CD, and EPR spectra were also recorded over the pH range 3–12 and in a Cu(II): peptide molar ratio 1 : 1 (Figure 2). The spectroscopic parameters are depicted in Table 4.

At acidic pH, CuH_2_L species predominate. Full spectroscopic characterization was not possible due to the low concentration of the complex and the overlapping with free Cu(II) and the CuL species distribution curves (Figure 1). Nevertheless, the log *K* value for the reaction Cu(II) + H_2_L ↔ CuH_2_L {log *K* = log *β* (CuH_2_L) − log *β* (H_2_L)} in our system (3.5) is comparable with literature data for the same type of reactions describing the formation of Cu(II)-imidazole bound complexes in the protected peptides -FKHV- (3.60), -MKHV- (3.70), and -IKQHT- (3.74) [25, 26]. Higher values were observed in the case of peptides -ELAKHA- (4.29), [11] -TESAHK- (4.47), and -TESHAK- (4.51) [7]. The difference is attributed to the stabilizing effect of the *γ*-carboxylate Glu residue in -ELAKHA-, -TESAHK-, and -TESHAK-. Such an effect is missing in our system.

**Scheme 2:**
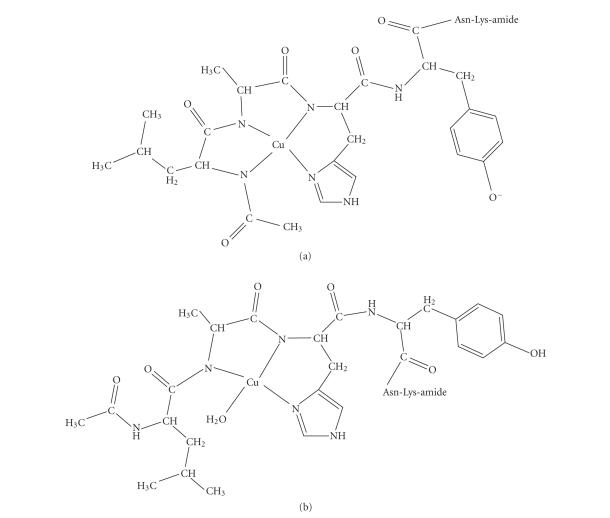
Possible structures of (a) CuH_−2_L (b) CuL.

The following species CuL is detected in neutral pH (Figure 1). Its very high concentration at pH ~ 7.3 which accounts for almost 98% of the total Cu(II) ion present and the wide pH range of existence (pH ~ 5–10.5) provide an indirect proof of its enhanced thermodynamic stability. CuL stoichiometry implies that a two proton ionization process took place cooperatively from the former species CuH_2_L (1N_im_ coordination mode) to CuL (3N coordination mode). Similar cooperativity has already been published for other peptide complexes containing histidyl residues [15, 17, 27]. The mean p*K*_*a*_ value for the ionization reaction CuH_2_L ↔ CuL + 2H^+^ is 5.75 (Table 3) and it is comparable to p*K*_*a*_ values for peptides with two amide nitrogens coordination (p*K*_*a*_ ~ 5.81–6.08) [23]. Additionally, the spectroscopic parameters of this species presented in Table 4 suggests a 3N{1N_im_, 2N_amide_} coordination mode according to literature data [7, 28–30]. The possible structure of this complex is depicted in Scheme 2 and it involves the equatorial coordination of the peptide through the imidazole and the amide nitrogens from His and Ala residues forming two stable, six- and five-membered chelate rings. The high-thermodynamic stability of complex CuL accounts for both the amide coordination cooperativity observed in this system and the suppression of the subsequent amide group binding.

In more basic solutions, a third proton was titrated (p*K*_*a*_ = 9.04) and species with stoichiometry CuH_−1_L were formed. The identical spectroscopic parameters of this species compared to CuL (Table 4) indicate that the source of this extra proton is not amide nitrogen. On the other hand, the p*K*_*a*_ value already mentioned is close to that of the phenolic –OH group of Tyr residue in the free ligand (p*K*_*a*_ = 9.52). This suggests the same binding modes of the studied hexapeptide in both CuL and CuH_−1_L complexes, differing only in the deprotonated but uncoordinated phenolic –OH group, in the case of the latter complex.

In contrast, the UV-Vis and EPR spectroscopic parameters of the next forming species CuH_−2_L are totally different (Table 4). A blue shift of the d-d band of about 54 nm (UV-Vis) the decrease of the g_||_ and concomitant increase of A_||_ (EPR) suggests a stronger ligand field around Cu(II) ion due to coordination of an additional nitrogen donor. Our spectroscopic data in Table 4 suggesting a 4N donor set are in excellent agreement with data concerning species presenting the same coordination mode in histidine-containing peptides Ac-FKHV-NH_2_ (*λ*_max_ = 528 nm, g_||_ = 2.185, A_||_ = 195 G), [25] Ac-GGHG-OH, (g_||_ = 2.20, A_||_ = 192 G) [31], and -TESAHK- (*λ*_max_ = 558 nm, g_||_ = 2.18, A_||_ = 198 G) [7]. The observed high p*K*_*a*_ value for the third amide ionization reaction (9.91) may be explained if we take into account that (a) deprotonation of an acetamido-group is less facilitated than that of the peptide bond [23] (b) the high stability of species CuL, (c) the negatively charged phenolic group of Tyr in species CuH_−1_L. Following the above conclusions, a possible coordination proposal for species CuH_−2_L is presented in Scheme 2. The metal ion coordination sphere includes the imidazole and three-deprotonated amide nitrogens, (the last belonging to the acetamido-group) forming three stable, six- and five-membered chelate rings.

Two more base consuming processes are observed in the pH range 10.5–12.0 corresponding to the formation of complexes CuH_−3_L and CuH_−4_L. Spectroscopic parameters (Table 4) for these species are almost identical to the ones of CuH_−2_L, implying the same coordination mode {1N_im_, 3N_amide_}. The p*K*_*a*_ value of the ionization reaction leading to CuH_−3_L is 10.53, exactly the same to that of the *ε*-NH_3_ group of Lys residue in the free ligand (Table 2). For the last dissociable proton (species CuH_−4_L), a p*K*_*a*_ value of 11.65 was calculated. An axially coordinated water molecule may be the best candidate of this additional proton (hydrolysis). In that case, a small red shift in the UV-Vis spectra and the decrease of the A_||_ (EPR) is expected [16, 32]. Unfortunately, the spectroscopic data (Table 4, Figure 2) provide no evidence on that. The only reasonable explanation remaining is the ionization and not coordination of the pyrrole N(1) of the imidazole ring. It is known [33] that the acidity of the pyrrole nitrogen increases upon coordination of a metal ion at the (N3) nitrogen atom. In the case of Cu(II):GlyGlyHis, a p*K*_*a*_ value of about 10.7 was calculated [34] while even lower values are observed when a second metal ion coordinates to this group. p*K*_*a*_ value of about 9.6 has been measured in the case of GlyHis:Cu(II) interaction and a tetranuclear structure with bridging imidazole was proposed [35]. Our value 11.65 is comparable to that corresponding to pyrrole ionization (and not coordination) in the system Cu(II):His 1 : 2 (11.7) [36] and Pd(GlyGlyHis-H_−2_) complex (11.3) [37]. To our knowledge, this is the first reported protected peptide presenting this type of ionization.

### 3.3. Ni(II) complexes

**Figure 3:**
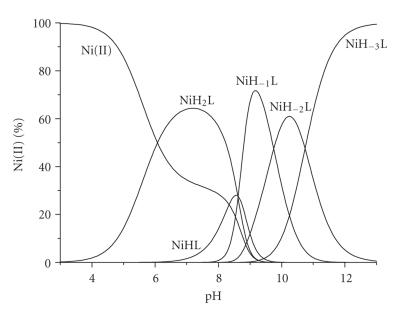
Species distribution diagram of Ni(II) complexes with -LAHYNK- (1 : 2), C_peptide_ = 2 mM.

The potentiometric study of the system Ni(II)-LAHYNK was performed in a 1 : 2 molar ratio in the pH range 4–11, in order to achieve shorter equilibrium times especially in neutral and alkaline pH values where sluggish reactions involving kinetically inert Ni(II) low-spin complexes are expected. The species distribution diagram is presented in Figure 3 while calculated stability data have already been presented in Table 3.

**Figure 4:**
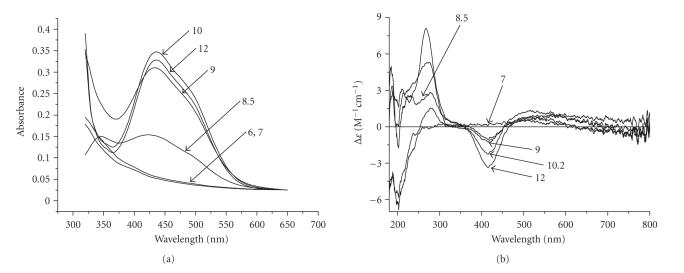
(a) UV/Vis and (b) CD spectra of the system Ni(II)/-LAHYNK- at various pH values.

The UV/Vis and CD spectra and the spectroscopic parameters at the pH range 4–12 at a metal:peptide ratio 1 : 2 are presented in Figure 4 and Table 4, respectively.

In general, the coordination properties of -LAHYNK- toward Ni(II) ions resemble the typical binding motifs of Ni(II) complexes with several peptides containing only one His residue in an internal position in their sequence [4, 10, 15, 38]. Five nickel complexes were detected in the pH range 4–12, namely NiH_2_L, NiHL, NiH_−1_L, NiH_−2_L, and NiH_−3_L.

The complex NiH_2_L is initially formed in the pH range 4.0–9.5 with an imidazole monodentate binding in a distorted octahedral geometry [10, 11, 39]. The log *K* value (3.21) for the reaction Ni(II) + H_2_L ↔ NiH_2_L shows a good agreement with literature values corresponding to monodentate coordination of imidazole N-donors (Ac-FKHV-NH_2_: 2.92, Ac-AKRHRK-NH_2_: 2.92, Ac-TESHAK-am: 3.20, Ac-ELAKHA:3.55) [4, 7, 11, 25].

On increasing pH, species NiH_2_L release a proton with a p*K*_*a*_ of 8.61. The p*K*_*a*_ value at 8.61 for the deprotonation process NiH_2_L → NiHL + H^+^ is comparable to the values for peptides with amide nitrogen coordination [10, 11, 15, 40]. The resultant complexes NiL cannot be detected spectroscopically due to overlap with other species in the pH range 7–9.5 and their low concentration. However, it is possible to characterize them on the basis of the stability constants corrected for protonation log K* [15]. The log K* value of these species (−11.63) are within the data range (−11.42−11.98) of similar complexes adopting an {N_im_, N_amide_} binding mode [4, 8, 23], supporting the coordination of Ni(II) ions through the imidazole and the amide nitrogen of the His residue forming a stable six-membered chelate ring.

**Scheme 3:**
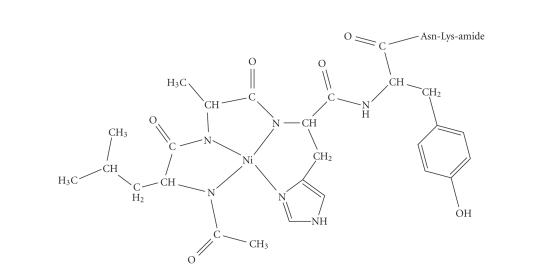
Possible structure of NiH_−1_L.

**Figure 5:**
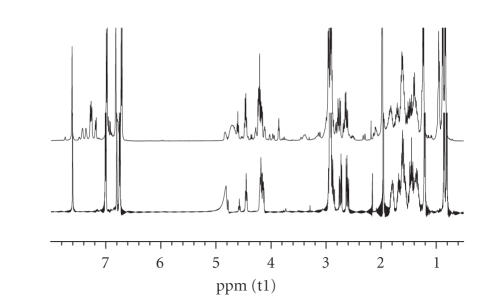
^1^H-NMR spectra (overlaid) of the free peptide (bottom) and of the Ni(II)-peptide solution (1 : 2) in D_2_O at pH* = 9.5.

The next three species NiH_−1_L, NiH_−2_L, and NiH_−3_L are detected above pH 8. The first one NiH_−1_L is derived from NiHL upon simultaneous release of two amide protons with an average p*K*_*a*_ value of 8.63 (Table 3). This process is very common in coordination chemistry of Ni(II)-peptides and the driving force for this is the formation of a stable square planar low-spin diamagnetic Ni(II) complex [15]. The data for NiH_−1_L, NiH_−2_L, and NiH_−3_L in Table 4 (d-d bands centered at *λ*_max_ = 422 − 446 nm the charge transfer bands N_im_ → Ni(II), and N_amide_ → Ni(II) detected at 329–336 nm) suggest the cooperative deprotonation and subsequent coordination of two more amide donors [7, 10, 11] saturating the equatorial plane of the Ni(II) ion in NiH_−1_L (Scheme 3). To strengthen more our binding proposal, the high-resolution ^1^H-NMR of the -LAHYNK-:Ni(II) system in 2 : 1 molar ratio was recorded in D_2_O at pH* = 9.5, providing an indirect proof of existence of the diamagnetic complex (Figure 5). Selected chemical shifts (*δ*, ppm) and chemical shifts differences (Δ*δ*, ppm) between free and Ni(II)-complexed peptide have already been presented in Table 1. Large chemical shifts differences are clearly observed for the *α*-H of the -L-A-H part of our peptide and of the C(2) –H of the imidazole ring confirming the proposed coordination mode.

Finally, the last two base-consuming processes which correspond to the formation of NiH_−2_L and NiH_−3_L species (p*K*_*a*_ values 9.75 and 10.74, resp.) are assigned to deprotonation (and no coordination) of Tyr –OH and Lys *ε*- NH_3_^+^ groups in the complexed peptide based on the similarity of the p*K*_*a*_ data for the free ligand (p*K*_*a*_ 9.52 and 10.53, resp.).

## 4. CONCLUSIONS

In this paper, the coordination properties of the N- and C-terminal blocked hexapeptide Ac-Leu-Ala-His-Tyr-Asn-Lys-amide (LAHYNK) representing the 80–85 fragment of histone H2B towards Cu(II) and Ni(II) have been investigated. Potentiometric and spectroscopic studies were used to establish the stoichiometry, stability and possible structures of all species formed in aqueous solutions.

In the case of Cu(II) complexes, it was found that at low pH values the initial binding site is the histidyl imidazole (CuH_2_L). The next species CuL formed at physiological pH values is a 3N complex {N_im_, 2N_amide_}. Its high thermodynamic stability is reflected in the cooperativity of the two amide ionization reactions and the suppression of the third leading to 4N species. The almost exclusive formation of the predominant species CuL in neutral media which accounts for almost 98% of the total metal ion concentration at pH 7.3 may imply that at pH values accessible by biological systems the sequence -LAHYNK- of histone H2B provides very efficient binding sites for metal ion. In more basic solutions, a third proton is titrated (p*K*_*a*_ = 9.04) and species with stoichiometry CuH_−1_L are formed. The coordination proposal for these is the same {N_im_, 2N_amide_} differing only in the deprotonated but uncoordinated phenolic –OH group. Finally, above pH ~ 10, 4N {1N_im_, 3N_amide_} species are detected in which acetamido-nitrogen donor saturates the equatorial plane of Cu(II). In the following two complexes CuH_−3_L and CuH_−4_L, a {1N_im_, 3N_amide_} binding is also evident following our experimental data, differing only in the deprotonated but uncoordinated phenolic –OH and pyrrole N(1) groups, respectively. It should be mentioned that in our knowledge, this is the first report of a protected peptide presenting this kind of ionization.

The coordination properties of -LAHYNK- toward Ni(II) ions resemble the typical binding motifs of Ni(II) complexes with several peptides containing only one His residue in an internal position in their sequence, that is, the monodentate imidazole binding in species NiH_2_L and the formation of typical low-spin diamagnetic 4N {1N_im_, 3N_amide_} complexes above pH ~ 8 (species NiH_−*n*_ L, *n* = 1 – 3) (Scheme 3).

No hydrolysis of the peptide was observed upon coordination to both Ni(II) and Cu(II) metal ions, in accordance to the same proposed mechanism of our group [10, 14] and others [9, 12] for such a process, implying the presence of a Ser- or Thr-containing an –OH group near the coordination sites [10, 14] to be important for the hydrolytic process. The formation of a 4N planar Ni(II) complex reported to be an important factor as well [9] is also present in our system.

## Figures and Tables

**Table 1:** Chemical shifts of ^1^H (*δ*, ppm) of free and Ni(II)-bound -TYTEHA-, in a metal:peptide ratio 1 : 1, at pH* 9.5.

-LAHYNK-	H_*α*_	H_*β*_	H_*γ*_	Others
Ac				1.95
^1^Leu	4.22 4.11 (−0.11)*	4.03	1.44	*δ*-CH_3_ *α*:0.81, *δ*-CH_3_ *β*:0.85
^2^Ala	4.19 3.38 (−0.81)*			CH_3_:1.20
^3^His	4.46 3.86 (−0.60)*	2.86, 2.92		H_2_ 7.61 7.25 (−0.36)*
				H_5_ 6.82 6.77 (−0.05)*
^4^Tyr	4.44 4.64 (+0.20)*	2.86, 2.92	2.14	H_2,6_ 6.97
				H_3,5_ 6.70
^5^Asn	4.60 4.60 (0.00)*	2.61, 2.73		
^6^Lys	4.16 4.23 (+0.07)*	1.67, 1.79	1.36	H_*δ*_:1.61, H_*ε*_:2.92

*Underlined the proton chemical shifts of the bound imidazole ring and C_a_-H (the chemical shift difference in parenthesis).

**Table 2:** Stability and ionization constants of the peptide -LAHYNK-.

Overall protonation constants (log *β*)^(a)^						
-LAHYNK-	HL	H_2_L	H_3_L	p*K*_His_	p*K*_Tyr_	p*K*_Tys_
	10.53 (2)	20.05 (2)	26.28 (3)	6.23	9.52	10.53

^(a)^ *β* = [H_*j*_L_*k*_]/([H]^*j*^ [L]^*k*^), standard deviations of the last digit are given in parenthesis.

**Table 3:** Stability constants of Cu(II) and Ni(II) complexes of -LAHYNK- at 25°C.

Stability constants of Cu(II) complexes (log *β*)^(a)^						
p*K* (*n*/*n* − 1)^(b)^	CuH_2_L	CuL	CuH_−1_L	CuH_−2_L	CuH_−3_L	CuH_−4_L
	23.55 (2)	12.06 (2)	3.02 (3)	−6.89 (2)	−17.42 (3)	−29.07 (4)
	2/0	0/−1	−1/−2	−2/−3	−3/−4	
	5.75^(c)^	9.04	9.91	10.53	11.65	

Stability constants of Ni(II) complexes (log *β*)^(a)^						

p*K* (*n*/*n* − 1)^(b)^	NiH_2_L	NiHL	NiH_−1_L	NiH_−2_L	NiH_−3_L	
	23.26 (2)	14.65 (4)	−2.60 (2)	−12.35 (2)	−23.09 (3)	
	2/1	1/−1	−1/−2	−2/−3		
	8.61	8.63^(c)^	9.75	10.74		

^(a)^ *β* = [M_*i*_H_*j*_L_*k*_]/([M]^*i*^[H]^*j*^[L]^*k*^) where M = Cu(II) or Ni(II), standard deviations of the last digit are given in parenthesis. ^(b)^For the ionization reaction MH_*n*_L ↔ MH_*n−1*_ L + H^+^, ^(c)^mean p*K* value for two protons release.

**Table 4:** Spectroscopic parameters of Cu(II) and Ni(II) complexes of -LAHYNK-.

Species	UV/Vis	CD	EPR	
	*λ*_max_ (*ε*/M^−1^ cm^−1^)	*λ*_max_ (Δ*ε*/M^−1^ cm^−1^)	A_||_ (G)	g_||_
Cu(II) complexes					

CuH_2_L (1N)	∗	331 (−0.33)^(b)^ 691 (0.11)^(a)^	∗	
CuL (3N)	591 (110)	338 (−0.98)^(b), (c)^ 637 (+0.30)^(a)^	170	2.230
CuH_−1_L (3N)	582 (94)	299 (+0.86)^(c)^ 339 (−0.68)^(b)^	173	2.231
		637 (+0.26)^(a)^		
CuH_−2_L (4N)	528 (120)	297 (1.00)^(c)^, 343 (−0.39)^(b)^	196	2.190
		475 (−0.54)^(a)^ 622 (+0.39)^(a)^		
CuH_−3_L (4N)	518 (140)	300 (+1.05)^(c)^, 348 (−0.15)^(b)^	196	2.180
		484 (−0.93)^(a)^ 614 (0.55)^(a)^		
CuH_−4_L (4N)	515 (150)	302 (+0.58)^(c)^ 346 (−0.17)^(b)^	196	2.180
		490 (−1.30)^(a)^ 624 (0.66)^(a)^		

Ni(II) complexes					

NiH_2_L (1N )	∗	∗	—	
NiHL (2N)	∗	329 (+0.34)^(b), (c)^	—	
		416 (−0.94)^(a)^		
NiH_−1_L (4N)	422 (84)	336 (+0.06)^(b), (c)^ 564 (+1.08)^(a)^	—	
		416 (−1.23)^(a)^		
NiH_−2_L (4N)	434 (140)	338 (−0.03)^(b), (c)^ 514 (+1.36)^(a)^	—	
		416 (−2.23)^(a)^		
NiH_−3_L (4N)	436 (130)	336 (+0.32)^(b), (c)^ 514 (+0.80)^(a)^	—	
		416 (−3.32)^(a)^		

^(a)^d-d transitions of metals, ^(b)^CT N_im_ → M, ^(c)^N^−^ → M, where M = Cu(II) or Ni(II). *These species could not be detected spectroscopically because of their low concentrations and/or overlap with others.
